# In Utero Cell Treatment of Hemophilia A Mice via Human Amniotic Fluid Mesenchymal Stromal Cell Engraftment

**DOI:** 10.3390/ijms242216411

**Published:** 2023-11-16

**Authors:** Yung-Tsung Kao, Chih-Ching Yen, Hueng-Chuen Fan, Jen-Kun Chen, Ming-Shan Chen, Ying-Wei Lan, Shang-Hsun Yang, Chuan-Mu Chen

**Affiliations:** 1 Department of Life Sciences, Ph.D. Program in Translational Medicine, National Chung Hsing University, Taichung 402, Taiwan; g106052319@mail.nchu.edu.tw; 2 Ph.D. Program in Tissue Engineering and Regenerative Medicine, National Health Research Institutes and National Chung Hsing University, Taichung 402, Taiwan; jkchen@nhri.edu.tw; 3 Department of Internal Medicine, Pulmonary Medicine Section, China Medical University Hospital, and China Medical University, Taichung 404, Taiwan; d5210@mail.cmuh.org.tw; 4 Department of Pediatrics, Department of Medical Research, Tungs’ Taichung Metroharbor Hospital, Wuchi, Taichung 435, Taiwan; fanhuengchuen@yahoo.com.tw; 5 Department of Rehabilitation, Jen-Teh Junior College of Medicine, Miaoli 356, Taiwan; 6 Institute of Biomedical Engineering and Nanomedicine, National Health Research Institutes, Miaoli 350, Taiwan; 7 Department of Anesthesiology, Ditmanson Medical Foundation Chia-Yi Christion Hospital, Chia-Yi 600, Taiwan; 06590@cych.org.tw; 8 Division of Pulmonary Biology, Cincinnati Children’s Hospital Medical Center, University of Cincinnati, Cincinnati, OH 45237, USA; bublelanwilliam@gmail.com; 9 Department of Physiology, Institute of Basic Medical Sciences, National Cheng Kung University, Tainan 70101, Taiwan; syang@mail.ncku.edu.tw; 10 The iEGG and Animal Biotechnology Center, National Chung Hsing University, Taichung 402, Taiwan; 11 Rong Hsing Research Center for Translational Medicine, National Chung Hsing University, Taichung 402, Taiwan

**Keywords:** hemophilia A mouse, in utero transplantation (IUT), stem cell therapy, amniotic fluid-mesenchymal stromal cells, clotting factor VIII (FVIII), FVIII inhibitors

## Abstract

Hemophilia is a genetic disorder linked to the sex chromosomes, resulting in impaired blood clotting due to insufficient intrinsic coagulation factors. There are approximately one million individuals worldwide with hemophilia, with hemophilia A being the most prevalent form. The current treatment for hemophilia A involves the administration of clotting factor VIII (FVIII) through regular and costly injections, which only provide temporary relief and pose inconveniences to patients. In utero transplantation (IUT) is an innovative method for addressing genetic disorders, taking advantage of the underdeveloped immune system of the fetus. This allows mesenchymal stromal cells to play a role in fetal development and potentially correct genetic abnormalities. The objective of this study was to assess the potential recovery of coagulation disorders in FVIII knockout hemophilia A mice through the administration of human amniotic fluid mesenchymal stromal cells (hAFMSCs) via IUT at the D14.5 fetal stage. The findings revealed that the transplanted human cells exhibited fusion with the recipient liver, with a ratio of approximately one human cell per 10,000 mouse cells and produced human FVIII protein in the livers of IUT-treated mice. Hemophilia A pups born to IUT recipients demonstrated substantial improvement in their coagulation issues from birth throughout the growth period of up to 12 weeks of age. Moreover, FVIII activity reached its peak at 6 weeks of age, while the levels of FVIII inhibitors remained relatively low during the 12-week testing period in mice with hemophilia. In conclusion, the results indicated that prenatal intrahepatic therapy using hAFMSCs has the potential to improve clotting issues in FVIII knockout mice, suggesting it as a potential clinical treatment for individuals with hemophilia A.

## 1. Introduction

Hemophilia A is a recessive X-linked genetic disorder characterized by the absence of factor VIII (FVIII) gene function. It affects about 80% of individuals in the general population with hemophilia [1]. Symptoms of hemophilia A include clotting defects, joint hemorrhage, and osteoporosis [2,3]. As of 2023, the estimated patient population with hemophilia globally has reached approximately 1,125,000, with a significant number of cases remaining undiagnosed [4]. The primary treatment for hemophilia A is replacement therapy, which involves the routine administration of FVIII concentrates. Currently, the cost of standard replacement treatment for hemophilia A is a significant burden, averaging USD 200,000–300,000 per patient per year. Moreover, this cost can increase by three-fold for patients with FVIII inhibitors [5]. However, this approach requires ongoing treatment. In recent years, emicizumab has represented a significant breakthrough. It is a bi-specific antibody that mimics FVIII to sustain the coagulation process. Nonetheless, it is important to note that regular injections are still necessary [6]. Gene therapy, a more recent development, offers alternatives. Viral vector gene therapy can achieve long-term therapeutic effects, but concerns have been raised regarding genome disruption or inflammatory responses associated with viral vectors. Non-viral vector gene therapy can also correct clotting problems without significant side effects [7]; however, it does not provide a permanent therapeutic effect. Another challenge in treating hemophilia A is the development of FVIII-neutralizing antibodies, known as FVIII inhibitors, in nearly 30% of patients [8,9]. This development renders the current treatments ineffective. Therefore, there is a pressing need for a new therapeutic strategy to address clotting issues in hemophilia patients and reduce the production of FVIII inhibitors.

Amniotic fluid mesenchymal stromal cells (AFMSCs) offer numerous advantages as a source of cell therapy, including their robust differentiation ability and stability [10,11,12,13]. These multipotent stromal cells have the ability to differentiate into various cell types, making them suitable for treating different cellular dysfunction diseases [14,15]. Additionally, AFMSCs exhibit low expression of major histocompatibility complex (MHC) class I molecules and lack the expression of MHC class II molecules [16], resulting in a reduced immune response upon transplantation. Given these attributes, AFMSCs present an ideal alternative to conventional gene therapy for the treatment of cell dysfunction diseases.

In utero cell transplantation (IUCT) using stem cells has shown promise in treating congenital genetic defects, including osteogenesis imperfecta [17,18]. The small size of the fetus allows for a lower number of stem cells to be used compared to transplantation in newborns or adults [19]. Moreover, the immature immune system of the fetus enables a more efficient participation of stem cells in tissue and organ development through IUCT, reducing the likelihood of rejection [20]. This makes therapeutic stem cell transplantation a viable option for addressing genetic diseases in the fetus.

Furthermore, infants with hemophilia are at clinical risk of intracranial hemorrhage (ICH) [21,22] and extracranial hemorrhage (ECH) [23], which can lead to neurological damage or even death. To prevent these hemophilia-related complications, it becomes necessary to initiate treatment before birth.

In this study, we utilized *FVIII* knockout (KO) mice as a model of hemophilia A and employed human AFMSCs (hAFMSCs), which can produce a FVIII [24] for xenogeneic in utero transplantation (IUT) to address coagulation disorders in *FVIII* KO mice. Our goal was for the hAFMSCs to contribute to fetal liver development, and in the liver environment, we expected them to differentiate into liver-related cells, thereby producing normal FVIII and reducing the presence of FVIII inhibitors in the bloodstream. After the birth of IUT-treated pups, we assessed the impaired coagulation time of the tail artery, FVIII activity, FVIII-neutralizing antibody levels in the blood, and the presence of human–mouse fusion cells in the liver to evaluate the feasibility of using human AFMSCs in IUT for treating hemophilia A.

## 2. Results

### 2.1. Characterization of Human AFMSCs

**Figure 1:**
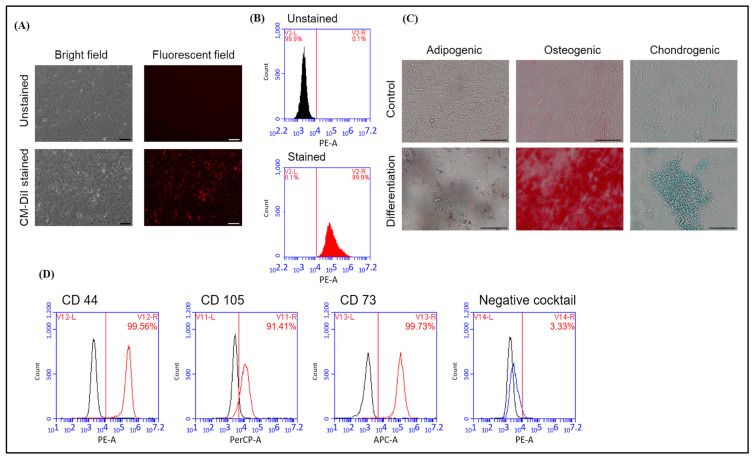
Characterization of hAFMSCs. (**A**) Bright field and fluorescent field showing the morphology of the unstained and CM-DiI-stained hAFMSCs. The original magnification is 100×, and the scale bar represents 100 μm. (**B**) The proportions of hAFMSCs stained and unstained with CM-DiI were detected via flow cytometry, and the results showed that approximately 99.9% of red fluorescent signals were detected in the CM-DiI-stained group. (**C**) Representative images of the three-lineage differentiation of hAFMSCs. Differentiated hAFMSCs were stained with Oil red O, Alizarin red S, and Alcian blue to detect adipocytes, osteocytes, and chondrocytes, respectively. The original magnification is 200×, and the scale bar represents 100 μm. (**D**) Characterization of hAFMSC CD surface markers. Positive and negative MSC markers were detected via flow cytometry. The percentage of positive CD markers was 99.56% for CD44, 91.41% for CD105, and 99.73% for CD73. The percentage of negative CD markers was approximately 3.33%. The negative cocktails included CD34, CD11b, CD19, CD45, and HLA-DR.

In order to track the hAFMSCs following their transplantation into the livers of hemophilia A mice, they were stained with the fluorescent dye CM DiI. Stained hAFMSCs were confirmed via fluorescence microscopy and flow cytometry (Figure 1A,B). The flow cytometry results showed a red fluorescence signal of approximately 99.9% for the stained hAFMSCs compared to 0.1% for the unstained hAFMSCs (Figure 1B).

In order to verify the multipotent differentiation capability of hAFMSCs, they were differentiated into trilineage cells of adipogenic, osteogenic, and chondrogenic states. The results showed that the hAFMSCs could successfully differentiate into adipocytes, osteocytes, and chondrocytes after induction with the appropriate induction medium (Supplementary Table S1 and Figure 1C). In order to characterize the surface markers of hAFMSCs, the cells were stained for the hMSC-positive CD markers CD105, CD73, and CD44 and the hMSC-negative cocktail CD markers CD34, CD11b, CD19, CD45, and HLA-DR. After staining, the cells were analyzed via flow cytometry. The results revealed that the hAFMSCs had 91% hMSC-positive CD markers and approximately 3.3% hMSC-negative cocktail markers compared to the isotype control (Figure 1D). These hAFMSCs were thus confirmed to exhibit conventional MSC features.

**Figure 2:**
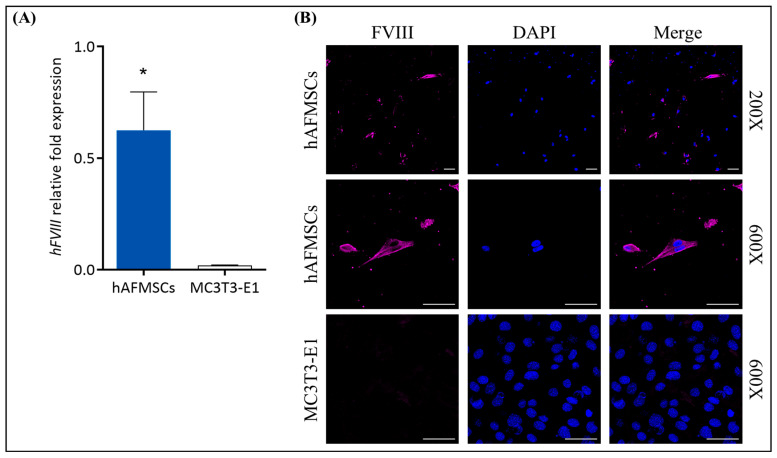
Assessment of FVIII expression in hAFMSCs. (**A**) The relative fold expression of *FVIII* mRNA was 0.6252 ± 0.1483 (n = 4) in hAFMSCs, as detected via quantitative RT-PCR (Q-RT-PCR), whereas in MC3T3-E1 cells, it was 0.0198 ± 0.0013 (n = 3), where *FVIII* could not be detected. *FVIII* mRNA expression was normalized to β-actin, with a *p*-value of 0.019 (* *p* < 0.05). (**B**) Immunofluorescence staining of hAFMSCs and MC3T3-E1 cells using a FVIII antibody and scanning with a confocal microscope revealed the presence of the FVIII signal in hAFMSCs, while no signal was observed in MC3T3-E1 cells. The original magnifications were 200× and 600×, with a scale bar of 50 μm.

Furthermore, we also examined the potential of hAFMSCs to express FVIII. We detected this via quantitative RT-PCR for mRNA expression and immunofluorescence staining for protein expression, using MC3T3-E1 cells as a negative control. The results indicated that FVIII mRNA was detectable in the hAFMSCs (n = 4), while there was no FVIII expression in the MC3T3-E1 control (n = 3) (Figure 2A). The immunofluorescence images illustrated that the hAFMSCs can produce FVIII, while there was no fluorescent signal in the MC3T3-E1 control (Figure 2B).

### 2.2. Detection of hAFMSCs in the Livers of IUT Fetuses

**Figure 3:**
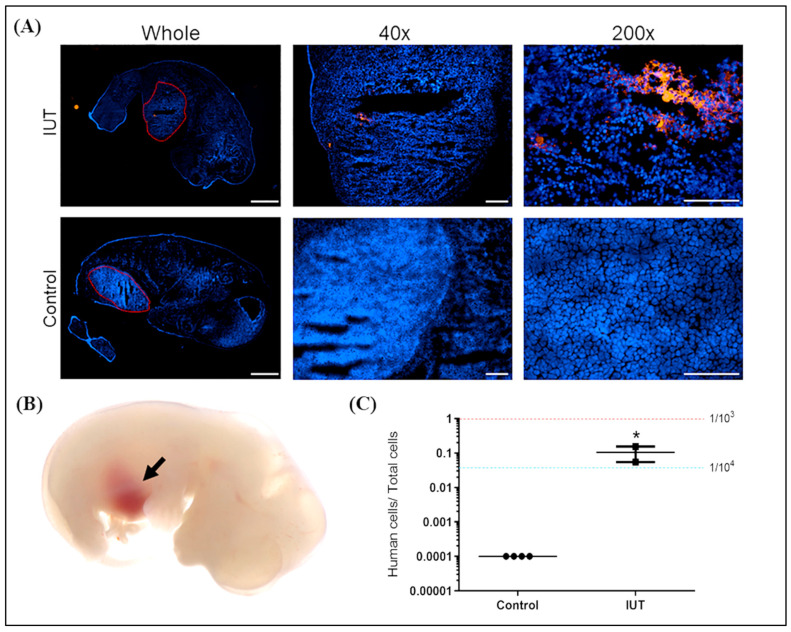
Analysis of human DNA and cells in a recipient mouse fetus. (**A**) Three days after the recipient fetus was injected with hAFMSCs, it was taken out from the uterus, and red fluorescent cells in the liver were observed under the microscope. The original magnifications were 4×, 40×, and 200×, and the scale bars were 1000 μm, 200 μm, and 100 μm, respectively. (**B**) Representative image of a fetal liver with brown color indicated by the black arrow after hAFMSC transplantation. (**C**) Quantitative PCR (Q-PCR) can detect human DNA in the liver of the IUT recipient fetus (n = 2), which was approximately one human cell in a thousand mouse cells, significantly higher than that of the controls (n = 4). The red and blue dashed lines represent the signal from one human cell among 10^3^ and 10^4^ mouse cells, respectively, with a *p*-value of 0.034 (* *p* < 0.05).

Fetuses were harvested at D17.5 of gestation after injection with CMDiI-stained hAFMSCs (Figure 3B). Fetal sections were examined with a fluorescence microscope. Red fluorescence was observed in the liver area of the IUT group, while no fluorescent signal was detected in the untreated control (Figure 3A).

Genomic DNA of the livers of IUT fetuses was extracted. A human-specific gene (hemoglobin subunit beta, *HBB*) was analyzed via Q-PCR, and a human/mouse ultra-conserved gene (transcription factor AP-2 alpha, *TFAP2A*) was used as an internal control. The standard control was the genomic DNA of one hAFMSC in 1000 and 10,000 mouse cells, indicated by red and blue dashed lines, respectively. The results indicated that human DNA could be detected in the IUT recipient livers at a ratio of approximately one human cell per 10,000 mouse cells (Figure 3C). In addition, the recipients exhibited significantly higher human cell signals compared to their untreated controls (*p* < 0.05).

### 2.3. Detection of Human Cells in the Livers of Recipient Mice

**Figure 4:**
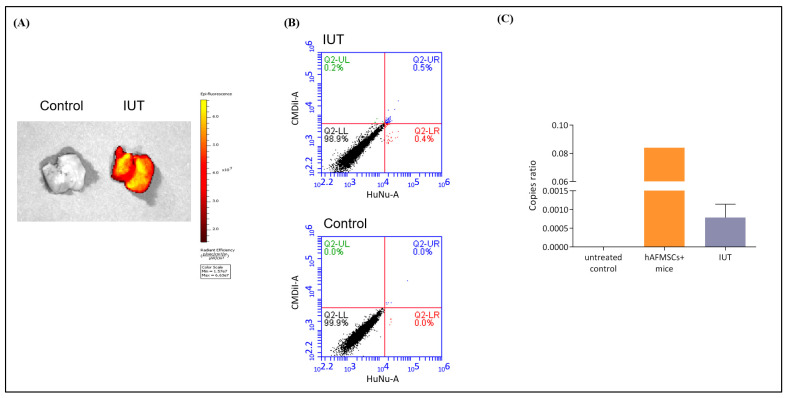
Human stem cells were detected in the livers of recipient mice. (**A**) Mouse livers were collected and detected for red fluorescence via an in vivo imaging system (IVIS). The results showed that the livers of the recipient mice exhibited obvious red fluorescence, while the livers of the control mice displayed no fluorescence. The scale is the calibrated units, which are expressed as “Photons per Second”. (**B**) Mouse liver single cells were stained with FITC-conjugated human nuclear (HuNu) antibody and analyzed via flow cytometry. Double-positive fluorescent cells were expressed in IUT-treated mice, whereas no fluorescent cells were detected in control mice. (**C**) The human-specific gene *HBB* was detected in the livers of recipient mice via digital droplet-PCR (ddPCR). Copy ratios were divided by *HBB* by the human–mouse conserved gene *TFPA2A*. The copy ratio of the IUT group was 0.000786 ± 0.000158 (n = 5); the standard control was the gDNA of hAFMSCs only and hAFMSCs diluted in mouse liver gDNA; the negative control was mouse liver gDNA.

The hAFMSC recipient mice were sacrificed at 13 weeks of age; the fluorescence of liver tissue was detected via IVIS and flow cytometry; the human gene was identified via ddPCR (Figure 4). Fluorescence signals in IUT recipient mouse livers were visible on IVIS images, whereas no fluorescent signals were detectable in the untreated controls (Figure 4A). Subsequently, the livers of recipient mice were lyzed into single cells and stained with FITC-conjugated human nuclear (HuNu) antibody, and the fluorescence signal of human cells was studied via flow cytometry. CMDiI and FITC combined signals were detected in the livers of recipients at 0.5%, whereas there were no fluorescence signals in the livers of untreated mice (Figure 4B).

To further confirm the presence of human cells in the livers of recipient mice, we used ddPCR to detect human genomic DNA signals. The human and mouse highly conserved *TFAP2A* gene was used as an internal control, and the human-specific *HBB* gene was used to detect human cells (Supplementary Table S2). The positive control was 100-fold diluted hAFMSC genomic DNA combined with mouse liver genomic DNA. The human gene signal was detected in the recipient mice, while it was not detected in the untreated mice (Figure 4C). The numbers of *HBB* droplet events in the untreated control, positive control, and IUT-treated groups were 0, 1112, and 8.6, respectively, while the *TFAP2A* internal control gene droplet events were 6806, 8854, and 7752, respectively (Supplementary Figure S1). Then, the droplet events were calculated using the Poisson distribution formula to obtain the copy number. The copy number ratio of *HBB* and *TFAP2A* in the IUT group was 0.00079 ± 0.00016 (n = 5) (Figure 4C). These data suggested that hAFMSCs can exist in the livers of IUT-treated mice.

### 2.4. Examination of the Human–Mousen Cell Fusion Phenomenon in Recipient Mice

**Figure 5:**
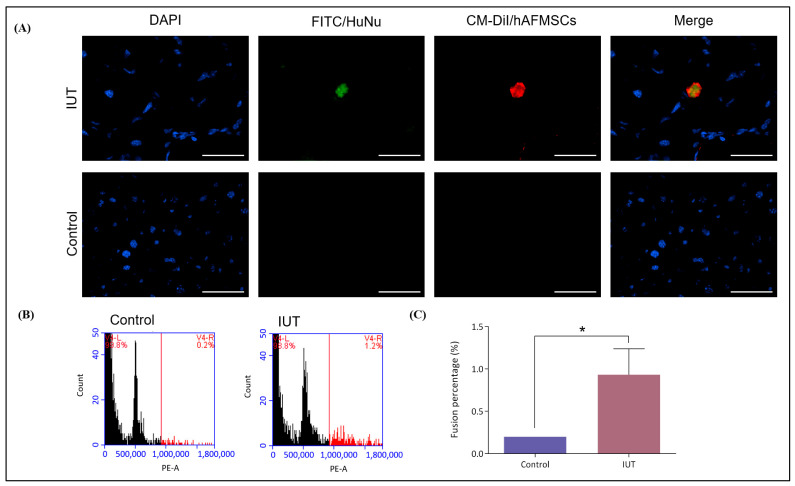
Evaluation of human–mouse cell fusion in the livers of recipient mice. (**A**) Liver tissue sections stained with DAPI and FITC-conjugated human nuclear (HuNu) antibodies. The livers of recipient mice exhibited double fluorescence colocalization compared with the livers of control mice, which displayed no fluorescence. The original magnification was 400×, and the scale bar was 50 μm. (**B**) Mouse liver single cells were stained with PI and examined via flow cytometry. The 2N and 4N chromosomes in the livers of IUT mice accounted for approximately 98.8%, and the > 4N chromosomes accounted for approximately 1.2%. The control mouse livers had only chromosomes 2N and 4N. (**C**) Chromosome numbers greater than 4N represent fusion cells. The fusion rate was 0.93 ± 0.18 (n = 3) in the IUT group and 0.20 ± 0.00 (n = 3) in the control group. There was a significant difference observed, with a *p*-value of 0.026 (* *p* < 0.05).

To validate cell fusion between transplanted human stem cells and host mouse cells, single-cell chromosome number and human cell colocalization were assessed in mouse liver tissues obtained from the 13-week-old recipient mice. Liver sections from the recipient mice were used to study human cells. Liver sections were stained with the FITC-conjugated HuNu antibody and observed under a fluorescence microscope. FITC and CMDiI signals were detected in the livers of recipient mice but not in the livers of untreated mice (Figure 5A). Furthermore, to confirm the chromosome number of fused cells, individual liver single cells were stained with PI and analyzed via flow cytometry. More than 2N and 4N chromosomes were detected in the IUT group. In contrast, only 2N and 4N chromosomes were detected in the control group (Figure 5B). The number of chromosomes over 4N in the IUT group was 0.93 ± 0.18, which was significantly higher than that in the control group, 0.20 ± 0.00 (*p* < 0.05) (Figure 5C). These data indicated that transplanted hAFMSCs could be retained in the livers of IUT mice and fused with mouse liver cells.

### 2.5. Observation of Human FVIII Protein in the Livers of Recipient Mice

**Figure 6:**
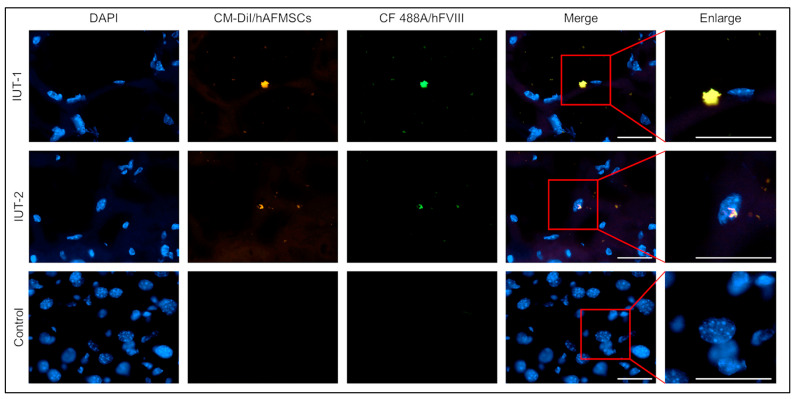
Identification of human cells and human FVIII expression in the livers of IUT recipient mice via immunofluorescence staining. The mouse liver tissue sections were stained with human FVIII (hFVIII) antibodies. The recipient mice livers can discern the hFVIII and transplanted human cells double fluorescent signal. In opposition, there were no hFVIII protein and human cells in the livers of untreated mice. The original magnification was 600×; the scale bar was 30 μm.

To confirm the presence of the human FVIII protein in the liver tissues of recipient mice, we utilized a human-specific FVIII antibody for detection. Our results demonstrated the co-localization of human FVIII (labeled with CF 488A) and transplanted human cells (labeled with CM-DiI) within the liver sections of the recipient mice, as visualized under a fluorescence microscope. Conversely, the untreated mice did not exhibit detectable levels of human FVIII protein or human cells (Figure 6). These findings provided compelling evidence that the hemophilia A mouse fetus accepted hAFMSCs during the gestation period, and subsequently, the transplanted cells were capable of producing human FVIII in the liver following birth.

### 2.6. Coagulation Problems in Hemophilia A Mice Were Corrected via hAFMSCs In Utero Therapy

**Figure 7:**
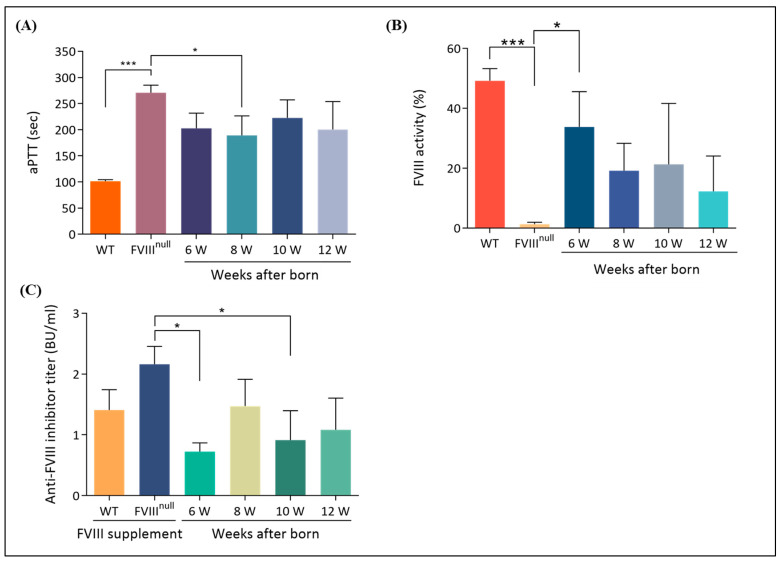
Improvement of coagulation problems in recipient mice. (**A**) The activated partial thromboplastin time (aPTT) test was used to evaluate the restoration of coagulation problems in recipient mice. The normal control was B6 mice, and the abnormal control was untreated F8KO mice. Clotting time was verified with citrated blood, which was lower in 6-, 8-, and 12-week-old recipient mice than in untreated F8KO mice. (**B**) FVIII activity was examined via a chromogenic assay. FVIII activity was detected in recipient mice and was significantly higher in 6-week-old mice than in untreated F8KO mice. (**C**) FVIII inhibitors were tested using a modified Nijmegen–Bethesda assay. Recipient mice were evaluated between 6 and 12 weeks of age, and blood samples were collected from the submandibular vein. The results demonstrated low levels of FVIII inhibitors during this period, with a significant decrease in 6-week-old mice compared to untreated F8KO mice. Bar graphs are presented as the mean ± SEM, and statistical analysis was performed using the one-way ANOVA and post-hoc LSD tests (* *p* < 0.05; *** *p* < 0.001).

In order to assess whether the FVIII deficiency problem was repaired after hAFMSC in utero transplantation, the mice were tested for aPTT, FVIII activity, and FVIII inhibitor levels after birth and every two weeks starting at six weeks of age. The results revealed that the aPTT value of untreated *FVIII* KO mice (270.71 ± 14.66 s; n = 7) was significantly higher than that of wild-type B6 mice (101.3 ± 3.03 s; n = 10) (*p* < 0.001). In contrast, the aPTT values of IUT-treated *FVIII* KO mice were 202.80 ± 29.01 s (n = 5), 189.13 ± 37.45 s (n = 8), 222.67 ± 34.38 s (n = 3), and 200.50 ± 53.5 s (n = 2) at 6, 8, 10, and 12 weeks of age, respectively, indicating a minor improvement in coagulation function compared with untreated *FVIII* KO mice (Figure 7A). The FVIII activity of the IUT group was examined from six to twelve weeks of age. The results showed that the FVIII activity of the WT mice was 49.24 ± 4.05% (n = 5) and that of the recipient mice was 33.80 ± 11.76% (n = 9), 19.17 ± 9.18% (n = 5), 21.28 ± 20.36% (n = 3), and 12.28 ± 11.80% (n = 3) at 6, 8, 10, and 12 weeks of age, respectively. In contrast, FVIII activity was 1.28 ± 0.64% in untreated FVIII KO mice (n = 11), and there was a significant difference between untreated FVIII KO mice and 6-week-old recipient mice (*p* = 0.020) (Figure 7B).

In order to verify whether *FVIII* KO mice produce FVIII inhibitors after treatment with hAFMSCs, the Nijmegen–Bethesda assay was used to test for FVIII inhibitor antibodies from 6 to 12 weeks of age. The data revealed that the IUT-treated recipient mice exhibited very low FVIII inhibitor developments, with Nijmegen–Bethesda assay values of 0.72 ± 0.15 (n = 9), 1.47 ± 0.44 (n = 5), 0.91 ± 0.49 (n = 3), and 1.08 ± 0.52 (n = 3) Bethesda units (BUs) at 6, 8, 10, and 12 weeks of age, respectively. The inhibitor value of untreated *FVIII* KO mice was 2.16 ± 0.30 (n = 3), and that of WT mice was 1.41 ± 0.33 (n = 3). There was a significant difference between untreated *FVIII* KO mice and 6-week-old recipient mice (*p* = 0.021) (Figure 7C).

## 3. Discussion

In this study, we introduced a novel in utero transplantation technique using hAFMSCs to address coagulation problems in *FVIII* KO mice with hemophilia. Our research yielded three main findings. Firstly, we confirmed the presence of transplanted FVIII-expressed human stem cells in the recipient mouse liver (Figure 2). This detection was made possible through fluorescence imaging and molecular analysis (Figure 4 and Supplementary Figure S1). Secondly, the transplanted hAFMSCs in recipient mouse liver tissues were observed to form hybrid cells between humans and mice. Chromosome analysis indicated the presence of more than 4N chromosomes (Figure 5). Lastly, the human FVIII protein was found in the treated mouse’s liver (Figure 6), and we observed an improvement in recipient *FVIII* KO mice throughout the 12-week study period. Additionally, the production of autologous FVIII antibodies remained relatively low, as evidenced using the aPTT test, FVIII activity assay, and assessment of FVIII inhibitors (Figure 7).

MSC-based therapies hold great promise for treating a range of diseases, including congenital defects [25], autoimmune diseases [26], cardiovascular diseases [27], and neurodegenerative diseases [28]. They have demonstrated effectiveness in tissue repair, organ regeneration, and various clinical applications [29]. The immunosuppressive properties and the ability to produce active FVIII make MSC transplantation a suitable approach for treating hemophilia A. MSCs currently used in preclinical research, animal studies, or human clinical trials are mainly derived from adipose tissue mesenchymal stromal cells (AD-MSCs) and bone marrow-mesenchymal stromal cells (BM-MSCs). However, the commonly used MSC sources have limitations, including donor dependence, heterogeneity, invasive procedures, and unsuitability for bleeding disorders [30]. To overcome these limitations, alternative MSC sources derived from medical waste during pregnancy, such as umbilical cord-mesenchymal stromal cells (UC-MSCs), placenta-derived mesenchymal stromal cells (PL-MSCs), and amniotic fluid-mesenchymal stromal cells (AF-MSCs), offer advantages such as non-invasiveness, ethical considerations, and the ability to reduce immune responses after transplantation. Additionally, when comparing AFMSCs with UC-MSCs and PL-MSCs, it is worth noting that AFMSCs can be isolated during gestation. This unique characteristic renders them suitable for the potential application of in utero therapy in the treatment of congenital disorders. Moreover, AFMSCs express low levels of major histocompatibility complex (MHC) class I molecules and no MHC class II molecules. In our study, we capitalized on these advantages and employed hAFMSCs to evaluate the efficacy of stem cell therapy in a *FVIII* knockout hemophilia A mouse model.

There are several routes for in utero cell-engrafted injection, including intravenous (i.v.), intraperitoneal (i.p.), intraplacental (i.pl.), and intrahepatic (i.h.) routes [31,32]. Long-term engraftment of transplanted stem cells is crucial for the success of cell-based therapies, including hemophilia A treatment. MSCs, in particular, require transplantation near the vasculature to efficiently release synthesized FVIII into circulation. In clinical trials, i.p. injections are commonly used due to their technical feasibility and perceived risk considerations [31,33], but recent advancements have explored i.v. injections in addition to i.p. delivery. Both the i.p. and i.v. routes can achieve stable long-term engraftment and transport of cells to the spleen, making them advantageous for hematopoietic disease treatment [31,34]. However, these routes may not be ideal for hemophilia therapy, as transplanted cells could migrate to other organs, reducing treatment efficacy [35,36]. Therefore, in this study, we employed i.h. injections to directly deliver hAFMSCs into fetal liver tissue. Our findings suggest that this approach enhances the therapeutic effect, demonstrating its potential for hemophilia treatment.

It is important to note that in clinical practice, IUT has shown promise in treating certain conditions. For instance, it has been successfully employed to address cases of osteogenesis imperfecta. In several instances, fetuses with this condition experience bone fractures during pregnancy, but IUT involving stem cell therapies has demonstrated improvements in these cases [17]. Furthermore, ongoing clinical trials are exploring the use of hematopoietic stem cells for the in utero treatment of alpha thalassemia in fetuses. This research aims to ameliorate fetal anemia during pregnancy. Additionally, as our knowledge of induced pluripotent stem cells (iPSCs) continues to expand, there is a growing belief that they could play a role in future in utero therapies [37]. Currently, virus vectors [38,39] and hematopoietic stem cells (HSCs) [40] are also commonly used in IUT treatment. Viral vectors can repair congenital genetic disorders but can pass through the blood–placental barrier and affect pregnant mothers [41]. MSCs transduced via genetically modified viral vectors are considered important candidates for the treatment of prenatal diseases [42]; they do not easily form teratomas after transplantation, and they encompass a strong renewal ability [43]. In addition, MSCs can reside in recipients for a long time via cell fusion [44,45]. Furthermore, the enhanced fusion of MSCs with target cells is achieved with greater efficiency through the expression of vesicular stomatitis virus glycoprotein (VSVG), thereby facilitating a regenerative therapeutic outcome [46]. In this experiment, we demonstrated that exogenous human AFMSCs can stably exist in the livers of recipients up to 12 weeks after birth in the form of human–mouse fusion cells and can continuously express active FVIII protein after in utero transplantation. The exact mechanism may be that FVIII produced by AFMSCs is secreted through extracellular vesicles or exosomes [47]. Although digital droplet PCR and flow cytometry quantification showed a low proportion of fusion cells (Figure 4 and Figure 5), due to the specificity of this treatment for hemophilia A, the low engraftment efficiency of transplanted cells may be sufficient to improve many symptoms of hemophilia A.

Although MSCs offer promising regenerative advantages, there are potential risks and challenges that must be carefully considered. A major concern lies in the transformation that may occur during cell culture, leading to mis-differentiation and potentially unfavorable outcomes. The choice of MSC passage and culturing conditions also plays a crucial role in influencing therapy outcomes. Long-term survival and potential complications necessitate careful attention and monitoring. Therefore, continuous surveillance for potential long-term effects is essential for patients undergoing MSC-based therapies. This emphasizes the importance of optimizing cell processing methods to enhance therapeutic effectiveness and patient safety [48,49]. The secretome of hAFMSCs plays an important role in treating hemophilia A in mice, and it can influence the fate of transplanted cells. Additionally, the role of MSC senescence and its impact on inflammaging should be carefully considered, suggesting the need for the careful selection of MSCs to ensure effective treatment outcomes. The analysis of mixed chimerism revealed promising engraftment and persistence of allogeneic cells in the treated mice, supporting their potential for long-term therapeutic benefit [50,51].

Patients with severe hemophilia A have less than 1% normal plasma FVIII activity. The standard of care for severe hemophilia A is prophylactic or scheduled intravenous injections of plasma-derived FVIII concentrates or recombinant FVIII products to prolong life. However, the short half-life of the FVIII protein necessitates frequent repeat injections, increasing the risk of infection, morbidity, and mortality [52]. Moreover, the development of anti-FVIII antibodies (FVIII inhibitors) in up to 30% of patients remains a major problem of FVIII replacement therapy [53]. In this study, FVIII inhibitor antibodies were examined from 6 to 12 weeks after birth, and the data revealed that IUT-treated recipient mice exhibited a very low postnatal development of FVIII inhibitors (Figure 7). In contrast to human fetal immune development, where the first mature T cells appear between 10 and 12 weeks of gestation, in mice, they only appear during the last few days of gestation [54,55]. Thus, we demonstrated that prenatal hAFMSC xenotransplantation at D14.5 prior to the appearance of T cells could overcome the FVIII inhibitor problem by inducing central tolerance at the fetal stage. In a clinical trial, fetuses with hemophilia can be identified before birth, allowing for the introduction of stem cells or FVIII gene-manipulated stem cells into the liver during the pregnant stage. The expectation is that the transplanted cells will differentiate into FVIII-producing cells within the liver environment. Furthermore, this therapeutic strategy aims to achieve immunological adaptation to prevent the development of inhibitory antibodies in hemophilia patients.

## 4. Materials and Methods

### 4.1. Animals

The animals used in this research were developed from a *FVIII* knockout (B6;129S-F8^tm1Kaz^/J) mouse strain purchased from the Jackson Laboratory (Bar Harbor, ME, USA). This mouse strain has less than 1% FVIII activity and is used as a hemophilia A model [3,5]. Eight-week-old C57BL/6 mice were obtained from BioLASCO Taiwan (Taipei, Taiwan) and used as a normal control group. All mice were housed under controlled temperature, humidity, and light conditions in a specific pathogen-free animal facility at National Chung Hsing University and had free access to a solid rodent chow diet and water. The animal study protocol was approved by the Institutional Animal Care and Use Committee of National Chung Hsing University, Taiwan (IACUC No. 104–045).

### 4.2. Culture of Human Amniotic Fluid Mesenchymal Stromal Cells

Human amniotic fluid mesenchymal stromal cells (hAFMSCs) were provided by Dr. Shiaw-Min Hwang of the Bioresource Collection and Research Center (Hsinchu, Taiwan). Cells were cultured in α-minimum essential medium (α-MEM) with 20% fetal bovine serum (FBS) (HyClone, Logan, UT, USA) and 4 ng/mL of basic fibroblast growth factor (b-FGF) (PeproTech EC, London, UK) and maintained in a 37 °C and 5% CO_2_ incubator [51]. Cells were passaged at a ratio of 1:4 when they were 80–90% confluent.

### 4.3. Characterized Human MSC CD Markers of hAFMSCs

hAFMSCs (0.5–1 × 10^7^) were trypsinized and labeled with an hMSC-positive cocktail containing CD105 PerCP-Cy5.5, CD73 APC, and CD44 PE antibodies (Becton Dickinson, BD, Franklin Lakes, NJ, USA). Subsequently, the hAFMSCs were labeled with an hMSC-negative cocktail containing CD34 PE, CD11b PE, CD19 PE, CD45 PE, and HLA-DR PE (BD) to identify potential contaminants. Next, the cells were incubated on ice for 30 min and washed twice with Perm/Wash buffer (BD). Finally, at least 10,000 cells were analyzed using an Accuri C6 plus flow cytometer (BD) to determine the surface markers and used CSampler™ Plus software version 1.0.27.1 (BD) to correct the fluorescent compensation [56,57].

### 4.4. In Vitro Differentiation Test of hAFMSCs

In order to confirm the differentiation capacity of hAFMSCs, cells were triggered to differentiate into three cell lineages, namely adipocytes, osteocytes, and chondrocytes. The cell populations were cultured to 100% confluency, trypsinized, seeded in 24 wells, and differentiated for 21 days using the appropriate induction medium (Supplementary Table S1). The medium was changed every three days during differentiation. The cultured cells were fixed with 4% paraformaldehyde (PFA) and stained with 0.5% Oil red O (Sigma-Aldrich, St. Louis, MO, USA) for adipocytes, 2% Alizarin red S (A5533; Sigma-Aldrich) for osteocytes, and 1% Alcian blue (B8438; Sigma-Aldrich) for chondrocytes, as previously described [57].

### 4.5. Detection of FVIII mRNA Expression in hAFMSCs via qRT-PCR

RNA was extracted from cell pellets using the Geneaid Biotech RNA Extraction Kit (Geneaid Biotech., Taipei, Taiwan); then, reverse transcription was performed using the cDNA Synthesis Master Mix (Thermo Fisher Scientific, Waltham, MA, USA). For the qRT-PCR reaction, a total volume of 10 μL was prepared, comprising 0.5 μL of cDNA, 0.5 μL of TaqMan probe (Topgen) or 0.4 μM of forward and reverse primers (see Supplementary Table S2), 5 μL of qPCRBIO Probe or SyGreen Blue Mix (PCR Biosystems, London, UK), and ddH_2_O. The PCR program consisted of an initial pre-denaturation at 95 °C for 3 min, followed by 40 amplification cycles (95 °C for 10 s and 60 °C for 30 s), and concluded with a melting curve analysis.

### 4.6. Immunofluorescence Staining

The 5 × 10^4^ cells were cultured on a culture chamber slide for 48 h and fixed with 4% PFA for 20 min. Further, the cells were permeabilized with 0.2% Triton X-100 for 10 min and blocked with blocking buffer (1% BSA, 0.3mM glycine in PBST) for 30 min. The cells were incubated at 4 °C overnight with primary mouse anti-hFVIII antibody (1:400 dilution; Millipore). Then, the cells were incubated with a secondary antibody conjugated with Alexa Fluor™ 647 (1:500 dilution; Thermo Fisher Scientific) and covered with a DAPI fluoromounting gel (SouthernBiotech, Birmingham, AL, USA). Fluorescent cell images were captured using a confocal laser scanning microscope (FV3000, Olympus, Tokyo, Japan).

### 4.7. In Utero Transplantation (IUT) Procedure

**Figure 8:**
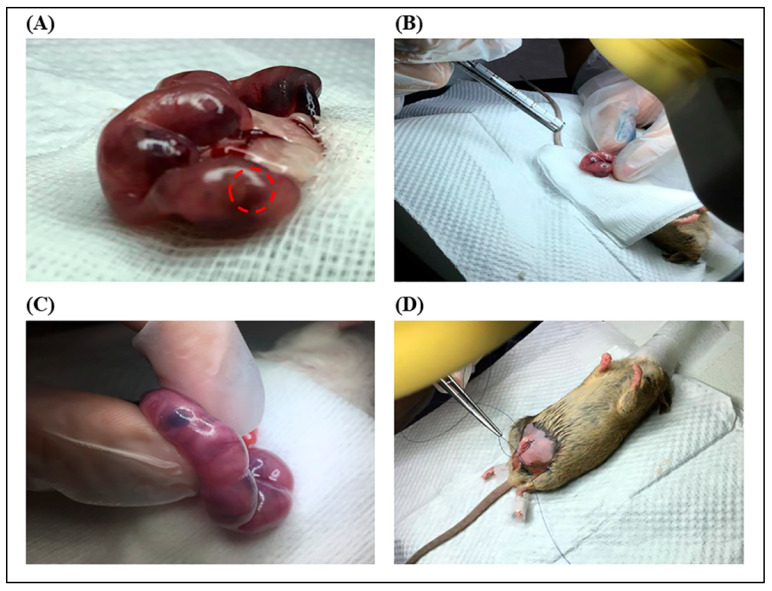
In utero transplantation (IUT) procedure. (**A**) A ventral incision was performed, and the uteri were removed from the mice. The represented fetal liver organ was marked by a red circle. (**B**) A total of 5 μL of indigo carmine–PBS containing 10^5^ red fluorescent labeled-AFMSCs was injected into the fetal liver. (**C**) A diagram of the liver stained blue with indigo carmine was observed after injection. (**D**) Schematic illustration of sheep catgut suturing an abdominal surgical wound.

In utero transplantation (IUT) was performed using *FVIII* KO female mice on day 14.5 of gestation. A total of 1 × 10^6^ hAFMSCs were trypsinized and stained with 3 μL of CMDiI prior to being used for IUT. After staining, the cells were resuspended in 50 μL of indigo carmine DPBS to allow for observations of the cells during IUT manipulation. The IUT operating procedures have been described below. First, the mice were anesthetized with 1.4% atm isoflurane; the abdominal hair was shaved; the skin was disinfected with povidone–iodine and 75% ethanol, and carprofen (China Chemical & Pharmaceutical Co., Taipei, Taiwan) was injected subcutaneously. Second, a ventral incision was made, and the entire uterus was pulled out and rinsed with warm saline. Next, 1 × 10^5^ CMDiI-stained hAFMSCs were injected into the liver of the fetus using a microsyringe (Hamilton Co, Anaheim, CA, USA). After IUT injection, it was important to confirm that the liver of the fetus was stained blue to ensure that the hAFMSCs were precisely injected into the liver. After confirmation, the uterus was returned to the abdominal cavity and rinsed with warm saline. Finally, the incision was sutured with catgut, and the mouse was put back into the cage for follow-up observation (Figure 8). The specify analyses were performed on each mouse were shown in the Supplementary Table S3.

### 4.8. Flow Cytometry Detection of Human Cells in Mice Livers

The recipient mice were sacrificed, and their livers were collected at 13 weeks of age. The liver tissues were minced into 3 mm fragments with a scalpel. Two milliliters of tissue lysis buffer (0.5 mg/mL of collagenase type IV; Worthington Industries, Columbus, OH, USA) and 0.05 mg/mL of DNase I in Hanks’ balanced salt solution (HBSS; Gibco, Paisley, Scotland, UK) were added to the liver fragments, and they were incubated at 37 °C for 30 min. The undigested tissue was filtered with 70 μm and 40 μm meshes and centrifuged at 1200× *g* for 5 min and discarded the supernatant. The cell pellet was resuspended in 6 mL of RBC lysis buffer and incubated at room temperature for 10 min. The cells were fixed with 4% PFA and stained with a monoclonal mouse anti-human nucleus (HuNu) antibody (MAB1281; Millipore, Waltham, MA, USA), which was conjugated with fluorescein isothiocyanate (FITC). After incubation at 4 °C overnight, the cells were washed with PBS, and the fluorescent signal was detected using an Accuri C6 plus flow cytometer (BD).

### 4.9. DNA Extraction and PCR

A total of 15–25 mg of liver tissue was used for genomic DNA extraction following the protocol of the Presto™ DNA/RNA/Protein Extraction Kit (Geneaid Biotech., Taipei, Taiwan). For DNA quantitative PCR (Q-PCR), the reaction contained 5 μL of ChamGE Probe qPCR Master Mix (#CGE-02, Topgen Biotech., Kaohsiung, Taiwan), 0.5 μL of TaqMan probes (Topgen) (see Supplementary Table S2), 100 ng of DNA sample, and the necessary volume of ddH_2_O to reach a total reaction volume of 10 μL. The sample mixture was loaded into 96-well plates and analyzed using the QuantStudio 6 Flex Real-Time PCR System (Thermo Fisher Scientific, Waltham, MA, USA). The PCR program was 95 °C for 3 min for pre-denaturation, followed by 40 amplification cycles of 95 °C for 10 s and 60 °C for 1 min.

### 4.10. Droplet Digital PCR (ddPCR)

The premix, which contains a 10 μL ddPCR Supermix for Probes (No dUTP, #1863024, Bio-Rad, Richmond, CA, USA), 1 μL of each TaqMan probe with FAM or VIC (Topgen) (Supplementary Table S2), 20–100 ng of DNA sample, and the necessary volume of ddH_2_O to reach a total volume of 20 μL, was prepared. The DNA mix was loaded onto the cartridge; 70 μL of droplet generation oil (Bio-Rad) was added; the cartridge was placed into the QX200 droplet generator (Bio-Rad) to generate the DNA–oil droplet. Then, the DNA–oil droplet was transferred to a 96-well plate and sealed with a PX1 PCR plate sealer (Bio-Rad), and PCR was executed using a T100™ thermal cycler (Bio-Rad). The PCR procedure was as follows: predenaturation at 95 °C for 10 min, 40 amplification cycles of 94 °C for 15 s and 60 °C for 1 min, and a final extension at 98 °C for 10 min, followed by chilling at 12 °C. Finally, the results were analyzed using a QX200 droplet reader QuantaSoft version 1.7.4.0917 (Bio-Rad).

### 4.11. In Vivo Imaging System (IVIS)

The livers of mice were washed with PBS, and the SpectrumCT In Vivo Imaging System (IVIS; Perkin Elmer, Waltham, MA, USA) was used to detect the fluorescent signal. The excitation filter was 535 nm, and the emission filter was 580 nm. The image was analyzed with IVIS software as previously described [58,59].

### 4.12. Histological Examination

Liver tissue sections of an appropriate size were fixed in 4% PFA and stored at 4 °C overnight. The fixed tissue was dehydrated with 30% sucrose solution and stored at 4 °C overnight. Subsequently, the tissues were embedded in a optimal cutting temperature (OCT) compound (Leica, Wentzler, Germany) and stored at −20 °C [60,61]. For the immunofluorescence assay (IFA), 5 μm-thick tissue sections were permeabilized with 0.5% Triton X-100 for 10 min and washed 3 times with PBS. The mouse anti-human HuNu antibody conjugated with FITC (1:100 dilution; Millipore) or mouse anti-hFVIII antibody (1:100 dilution; Millipore) was used to stain the tissue sections at 4 °C overnight. After washing 3 times with PBS, the anti-hFVIII antibody was incubated with a secondary antibody conjugated with CF 488A (1:200 dilution; Millipore). Finally, the tissue sections were stained with DAPI (1:1000 dilution; BioPioneer Co., Taipei, Taiwan). IFA images were examined using a fluorescence microscope (Lionheart™ FX Automated Microscope, BioTek Instruments, Winooski, VT, USA).

### 4.13. Cell Fusion Chromosome Test

Single liver cells were washed with PBS and fixed with 70% ethanol at −20 °C overnight. The cells were treated with 0.5% Triton X-100 (Amresco Inc., Solon, OH, USA) and 0.05% RNase A buffer (GMbiolab Co., Taichung, Taiwan) at 37 °C for 40 min and washed with PBS. Then, the cells were stained with 50 μg/mL of propidium iodide (PI: #25535–16–4, Sigma-Aldrich) at 4 °C for 20 min and analyzed using an Accuri C6 plus flow cytometer (BD).

### 4.14. The activated Partial Thromboplastin Time (aPTT) Assay

Mice were anesthetized with 1.4% atm isoflurane, and blood was collected from the submandibular vein. Blood was mixed 9:1 with 3.2% sodium citrate, and the citrated blood was added to the Coag Dx Analyzer (IDEXX, Westbrook, ME, USA) for the clotting assay. The time until blood clotting was recorded as the aPTT value, and if the aPTT value was out of range, it was recorded as 300 s [6].

### 4.15. The FVIII Activity Assay

FVIII activity was tested via FVIII chromogenic assays (Siemens, Marburg, Germany) as described [6]. Briefly, a 10 μL plasma sample was diluted with 90 μL of 0.9% NaCl solution, and 20 μL was added to 96-well plates. Then, 20 μL of factor IXa reagent and factor X reagent were added to each well, and the plates were incubated at 37 °C for 90 s. Then, 100 μL of substrate reagent was added to each well, and the plates were incubated for 60 s. Finally, 20 μL of 20% acetic acid was added to each well to terminate the reaction, and the absorbance at OD 405 nm was read. The FVIII activity of the sample was calculated against the standard curve, which was made via serial dilutions of the standard human plasma (Siemens) according to the instructions of the kit.

### 4.16. The FVIII Inhibitor Antibody Assay

The control group of mice were administered FVIII-neutralizing antibodies via weekly intraperitoneal injections of 600 IU/kg of Simoctocog alfa Nuwiq^®^ recombinant human coagulation factor VIII (Octapharma, Lachen, Switzerland) for a duration of four weeks. Subsequently, one week later, a boost of 1200 IU/kg was administered, and mouse plasma samples were collected the following week.

The plasma sample was heat deactivated at 56 °C for 30 min, and a 10 μL plasma sample was diluted with 10 μL of IB-BSA (imidazole-buffered bovine serum albumin, pH = 7.4, 50 mM imidazole). Then, IB-PNP (imidazole-buffered pooled normal plasma, pH = 7.4, 100 mM imidazole, FVIII 95–105%) was added to the sample, and it was incubated at 37 °C for 2 h and placed at 0 °C for 10 min to terminate the reaction. Finally, the FVIII chromogenic assay (Siemens) was used to determine FVIII activity relative to the standard IB-PNP control. To calculate the Bethesda unit, the FVIII residual activity (RA%) of the sample was divided by the standard control (sample mix/control mix × 100), and the formula [(2-log RA%)/0.30103] × dilution factor was used to obtain the Bethesda unit.

### 4.17. Statistical Analysis

All data were expressed as the mean ± standard error of the mean (SEM), and the statistical analyses were performed using the Student’s *t*-test or one-way ANOVA and post-hoc LSD test, and a *p* < 0.05 indicated significant differences.

## 5. Conclusions

In this study, we successfully developed a novel in utero cell therapy via human amniotic fluid mesenchymal stromal cell engraftments for hemophilia A mice. The transplanted human cells were detected as fusion cells in the recipient livers at a ratio of approximately one human cell per 10 thousand mouse cells and produced human FVIII protein in the livers of IUT-treated mice. The coagulation problems in hemophilia A mice significantly improved after birth and continued to display improvements throughout the 12-week observation period. FVIII activity reached its peak at 6 weeks of age, while FVIII inhibitors remained relatively low in hemophilic recipient mice. Based on these findings, we concluded that prenatal intrahepatic hAFMSC therapy has the potential to effectively address clotting issues in FVIII KO mice and could be a promising clinical treatment for patients with hemophilia A disease.

## Data Availability

The data presented in this study are available on request from the corresponding author.

## Supplementary Materials

The following supporting information can be downloaded at: https://www.mdpi.com/article/10.3390/ijms242216411/s1.
