# Supplementary material for: In Utero Cell Treatment of Hemophilia A Mice via Human Amniotic Fluid Mesenchymal Stromal Cell Engraftment
Source: Int J Mol Sci. 2023 Nov 16;24(22):16411. doi: 10.3390/ijms242216411 (PMC10670993; doi:10.3390/ijms242216411)
Supplement: Supplementary file 1 [file ijms-24-16411-s001.zip › ijms-2649050-supplementary.pdf]

Supplementary Table S1. Preparation of hAFMSCs trilineage differentiation media

Reagent	Working concentration
Adipogenic differentiation medium	
Dexamethasone	1 µM
Insulin	10 µg/ml
Isobutylmethylxanthine (IBMX)	0.5 mM
Indomethacin	10 µM
Fetal bovine serum (FBS)	10%
Penicillin/Streptomycin (P/S)	1%
Osteogenic differentiation medium	
Dexamethasone	0.1 µM
L-ascorbic acid 2-phosphate	0.2 mM
β-glycerophosphate	10 mM
Fetal bovine serum (FBS)	10%
Penicillin/Streptomycin (P/S)	1%
Chondrogenic differentiation medium	
Dexamethasone	0.1 µM
Insulin	10 µg/ml
Transferrin	5.5 µg/ml
Sodium selenite	7 ng/ml
L-ascorbic acid 2-phosphate	0.2 mM
Transforming growth factor-β1	10 ng/ml
Fetal bovine serum (FBS)	10%
Penicillin/Streptomycin (P/S)	1%

Supplementary Table S2. The information of Taqman probe and primer sequences for PCR and ddPCR used in this study

Gene name	Sequence (5’ to 3’)	5’ Fluorescent reporter	3’ Quencher	Tm (˚C)	Amplicon size (bp)
HBB	F: 5’- CTGTGTTCACTAGCAACCTC-3’<br>R: 5’- CTGTCTTGTAACCTTGATACCAA-3’<br>P: 5’- ATCCACGTTCACCTTG-3’	VIC	MGB	60	79
TFAP2A	F: 5’- CAATGGGTTCAGCTGCTTATTG-3’<br>R: 5’- CCCAGGCGTATTTTTGTTCT-3’<br>P: 5’- AATTGCCAAGGGA-3’	FAM	MGB	60	111
FVIII	F: 5’- ACCAGCATGTATGTGAAGGAGTTC-3’<br>R: 5’- CCACAGGTGTGAAGGAGTCTTG-3’<br>P: 5’- AGCAGTCAAGATGGC-3’	FAM	MGB	60	124
β-actin	F: 5’- ACCAGCATGTATGTGAAGGAGTTC-3’<br>R: 5’- CCACAGGTGTGAAGGAGTCTTG-3’			60	143

Supplementary Table S3. The information of specify analyses were performed on each mouse in this study

Recipient mice number	Analyses
No.1	aPTT, FVIII activity test, FVIII inhibitor test, ddPCR test
No.2	aPTT, FVIII activity test, FVIII inhibitor test, ddPCR test
No.3	aPTT, FVIII activity test, FVIII inhibitor test
No.4	aPTT, FVIII activity test, FVIII inhibitor test
No.5	aPTT, FVIII activity test, FVIII inhibitor test
No.6	aPTT, FVIII activity test, FVIII inhibitor test
No.7	aPTT, FVIII activity test, FVIII inhibitor test, ddPCR test, Cell fusion test
No.8	aPTT, FVIII activity test, FVIII inhibitor test, ddPCR test, Cell fusion test
No.9	aPTT, FVIII activity test, FVIII inhibitor test, ddPCR test, Cell fusion test

(A)

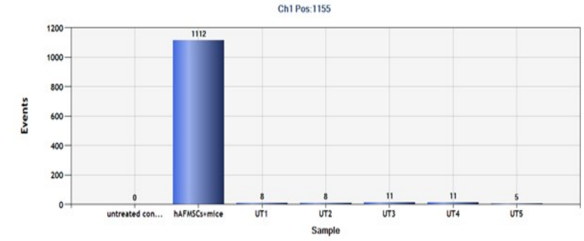

(B)

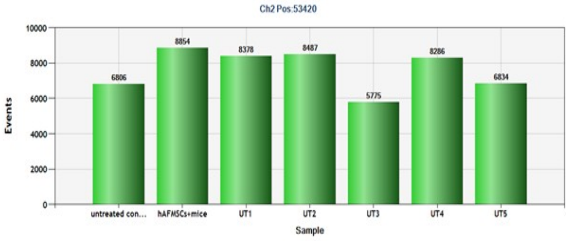

(C)

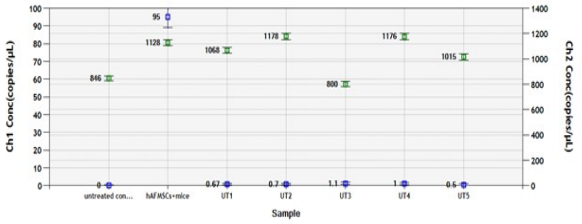

(D)

Analysis	Droplet (events)		Copies/μL		
Gene / Groups	*HBB*	*TFPA2A*	*HBB*	*TFPA2A*	Ratio
Untreated mice	0	6806	0	846	0
hAFMSCs +mice	1112	8854	94.8	1128	0.084043
IUT 1	8	8378	0.67	1068	0.000627
IUT 2	8	8487	0.7	1178	0.000594
IUT 3	11	5775	1.1	800	0.001375
IUT 4	11	8286	0.99	1176	0.000842
IUT 5	5	6834	0.5	1015	0.000493

Supplementary Figure S1. The droplet events and copy numbers of *HBB* and *TFPA2A* gene amplicons detected by the ddPCR. (**A**) The *HBB* gene droplet events. (**B**) The *TFPA2A* gene droplet events. (**C**) The copy numbers of *HBB* (blue spots) and *TFPA2A* (green spots) calculated by Poisson distribution formula. (**D**) The *HBB* and *TFPA2A* droplet events and copy number ratios.
